# Sentinel Lymph Node Biopsy Should Be Included with the Initial Surgery for High-Risk Ductal Carcinoma-In-Situ

**DOI:** 10.1155/2014/624185

**Published:** 2014-10-29

**Authors:** Ern Yu Tan, Z. W. Joseph Lo, Chuan Han Ang, Christine Teo, Melanie D. W. Seah, Juliana J. C. Chen, Patrick M. Y. Chan

**Affiliations:** Department of General Surgery, Tan Tock Seng Hospital, 11 Jalan Tan Tock Seng, Singapore 308433

## Abstract

*Background*. A proportion of those diagnosed preoperatively with ductal carcinoma-in-situ (DCIS) will be histologically upgraded to invasive carcinoma. Repeat surgery for sentinel lymph node (SLN) biopsy will be required if it had not been included with the initial surgery. We reviewed the outcome of SLN biopsy performed with the initial surgery based on a preoperative diagnosis of DCIS and aimed to identify patients at risk of histological upgrade. *Methods*. Retrospective review of 294 consecutive female patients diagnosed with DCIS was performed at our institute from January 1, 2001, to December 31, 2008. *Results*. Of the 294 patients, 132 (44.9%) underwent SLN biopsy together with the initial surgery. The SLN was positive for metastases in 5 patients, all of whom had tumours that were histologically upgraded. Histological upgrade also occurred in 43 of the 127 patients (33.9%) in whom the SLN was negative for metastases. On multivariate analysis, histological upgrade was more likely if a mass was detected on mammogram, if the preoperative diagnosis was obtained with core biopsy and if microinvasion was reported in the biopsy. *Conclusion*. Patients in whom a preoperative diagnosis of DCIS is likely to be upgraded to invasive carcinoma will benefit from SLN biopsy being performed with the initial surgery.

## 1. Introduction

Ductal carcinoma-in-situ (DCIS) of the breast, by definition, does not invade beyond the basement membrane and nodal metastasis is not expected. However, axillary nodal involvement has been reported in 2% to 13% of DCIS [1, 2]. The axillary nodes were seldom evaluated in the past as potential morbidity from full axillary nodal dissection (ALND), where the level I and II nodes are removed, and even from axillary sampling, outweighed the possibility of finding nodal disease that would change treatment recommendations [3]. But with the widespread adoption of the simpler and less morbid technique of sentinel lymph node (SLN) biopsy, there have been efforts to reevaluate the role of nodal evaluation in DCIS. Nodal disease is often an indication of microinvasive or invasive foci present within the tumour and is rare in pure DCIS tumours, where it may signify the presence of occult microinvasion. The presence of nodal disease may warrant consideration for more aggressive systemic therapy in view of the potentially higher risk of disease recurrence and systemic metastasis [4, 5]. Still, the incidence of nodal metastases is too low to justify SLN biopsy in all DCIS tumours.

Sentinel lymph node biopsy is indicated in the 10% to 20% of DCIS tumours in which foci of invasive carcinoma are detected after surgical excision and analysis of the entire lesion [6, 7]. Such histological upgrading has become more common now that the preoperative diagnosis of DCIS is often made on core biopsy. Most surgeons will include a SLN biopsy when performing a mastectomy for DCIS since access to the SLN lies within the operative field and also because nodal evaluation after a mastectomy often entails ALND as the SLN can seldom be successfully identified due to the disruption of lymphatic drainage to the axilla. On the other hand, with wide local excision (WLE), a separate incision in the axilla is required for SLN biopsy and even a WLE performed in the upper outer quadrant does not interfere with successful SLN identification at a later setting. Including a SLN biopsy with the initial WLE may not reduce the reoperation rate as much as with a mastectomy, since up to 20% of these women will need a second surgery because of inadequate surgical margins [8]. Nevertheless, having a SLN biopsy done together with the first surgery will undoubtedly save those women in whom a preoperative diagnosis of DCIS is upgraded to invasive carcinoma on final histology from a second surgery. Avoiding a second surgery saves time and costs, and more importantly, it reduces psychological stress and facilitates recovery. Completing all necessary surgery in a single setting is particularly important in our society where the need for a second surgery is often perceived as a failure of the initial one.

At our institute, since 2006 when SLN biopsy was adopted as the standard of care, SLN biopsy was included in the initial surgery if it involved a mastectomy. Most surgeons performed WLE alone for a preoperative diagnosis of DCIS, while some surgeons selectively offered SLN biopsy together with the initial WLE if the likelihood of histological upgrade to invasive carcinoma was deemed to be high. In this study, we have reviewed our data to evaluate the outcome of SLN biopsy performed together with the initial surgery for a preoperative diagnosis of DCIS. In addition, we sought to identify the subgroup of patients who were at risk of a histological upgrade to invasive carcinoma since they would benefit from having SLN biopsy performed at the time of the initial surgery.

## 2. Methods

A retrospective review of 348 consecutive female patients who were diagnosed preoperatively with DCIS was performed at our institute over an 8-year period from January 1, 2001, to December 31, 2008. This study had Ethics Committee Approval (D/10/029). Patients who did not undergo surgery, either because they had defaulted treatment or because they were deemed medically unfit, were excluded, as were those in whom definite foci of microinvasion had been reported on the biopsy specimen.

Preoperative diagnosis was routinely obtained via a core biopsy in our practice. Biopsy was performed free hand for palpable lesions, and under image guidance, preferably with breast ultrasound, for nonpalpable ones. Mammographically guided biopsies were reserved for sonographically occult lesions. Ultrasound-guided biopsy was performed by dedicated breast interventional radiologists with a 14-gauge Bard needle under local anaesthesia and usually 4 to 6 cores (average of 5) were obtained; more cores were taken if the target lesion was vague or large. Stereotactic vacuum assisted biopsy was performed with an 11-gauge needle and usually 6 to 12 cores (average of 10) were obtained and immediately imaged to confirm the presence of microcalcifications. Further cores were obtained if the initially visualised microcalcifications were absent in the biopsy specimens.

Surgery was performed by a total of 8 specialist breast surgeons during this period. From 2001 to 2005, SLN biopsy was performed selectively, according to surgeon preference and experience. Some surgeons performed axillary sampling together with a mastectomy during this period. From 2006 onwards, SLN biopsy was adopted as the standard of care and was routinely performed together with a mastectomy. SLN biopsy was also performed together with the initial WLE if microinvasive foci were reported in the biopsy specimen. Some surgeons offered SLN biopsy together with the initial WLE when features such as high nuclear grade and necrosis were present in the biopsy specimen. All SLN biopsies were performed with blue dye alone. Two mL of undiluted patent V blue dye was injected via a subareolar route after induction and 5 minutes of manual massage followed. The SLN was identified as a blue-coloured level I node with blue lymphatic channels leading up to it. SLNs were submitted for intraoperative frozen section analysis or for routine histological analysis only according to surgeon preference. The number and size of SLNs submitted for analysis were recorded and each node was serially sliced at 2 to 3 mm gross intervals. For intraoperative frozen section analysis, each slice was further sectioned at intervals of 40 *μ*m to obtain 3 sections, which are stained in haematoxylin and eosin (H&E) and are examined using routine light microscopy. The entire SLN was then formalin-fixed, processed, and paraffin-embedded to obtain permanent sections for final analysis, where additional 1 to 6 levels of each slice of the SLN were examined. The presence or absence of tumour deposits was recorded according to current established cancer reporting guidelines provided by the College American Pathologists (October 2009). The SLN was considered positive when tumour deposits of more than 0.2 mm (micro- and macrometastasis) were present and negative when no tumour cells were detected or when only single tumour cells or tumour cell clusters not more than 0.2 mm (isolated tumour cells) were present. Molecular tests such as immunohistochemistry and quantitative real-time PCR were not routinely performed for H&E-negative sections, although immunohistochemistry with anticytokeratin antibodies was used in certain instances to verify the presence of metastasis.

Adjuvant therapy was recommended according to current treatment guidelines. Whole breast irradiation was recommended for those who underwent WLE; an additional boost to the tumour bed was not routinely administered unless the surgical margins were inadequate and if the patient had refused further surgery. Chemoprevention with Tamoxifen was recommended for all with hormone responsive tumours. Systemic chemotherapy and targeted therapy were recommended in those with invasive carcinoma according to guidelines.

Histological upgrade was defined as the instance where histological analysis of the surgical specimen revealed microinvasive or invasive foci within a tumour which had been preoperatively diagnosed as being DCIS. Histological upgrade to invasive carcinoma was correlated with standard clinicopathological parameters, including patient age, clinical presentation, radiological features, pathological tumour size, nuclear grade, necrosis, possible microinvasive foci, and hormone receptor (oestrogen receptor (ER) and progesterone receptor (PR) status). Correlation analyses were performed using either the Chi square test or Fisher's exact test where appropriate. Correlation with tumour grade was evaluated using the Chi square test for trend. All univariate analyses were performed with GraphPad Prism Version 4 (GraphPad Software Inc., San Diego, CA). Cox proportional hazard regression model was used to identify independent predictors of histological upgrade. This was performed using the Stata package release 8.1 (Stata Corporation, 4905 Lakeway Drive, College Station, Texas 77845, USA). A full model was first created to include all potentially important explanatory variables. At each step, the variable with the smallest contribution to the model was removed, until a final backward stepwise model was obtained. A 2-tailed *P* value test was used in all analyses and a *P* value of less than 0.05 was considered statistically significant.

## 3. Results

A total of 294 patients were included in the final analysis. One hundred and twenty-eight patients (43.5%) presented with clinical symptoms, 110 with a breast lump and 18 with nipple discharge. Median patient age was 51 years (ranging from 30 years to 91 years). Ethnic distribution reflected that of the general population (Table 1), with Chinese women making up the large majority. DCIS was diagnosed on core or vacuum assisted biopsy in 79.3% (233 of 294) of patients. A mastectomy was performed in 126 patients (42.9%) and a WLE in the remaining 168 patients. Median follow-up was 82.0 months (ranging from 3.7 to 143.0 months).

One hundred and thirty-two patients (44.9%) underwent SLN biopsy at the time of the initial surgery, which involved a mastectomy in 70 patients and a WLE in the other 62. The SLN was positive for metastases in 5 patients (3.8%), all of whom were found to have invasive ductal carcinoma on final histology. The invasive tumour was classified as T1a in 3 patients (tumour size ranging from 1 mm to 4 mm), as T1c (tumour size 19 mm) in 1 and as T2 (tumour size 25 mm) in the remaining patient. Micrometastatic deposits (detected on H&E) were found in 2 patients, and macrometastases were present in the other 3. Median age of this group of patients was 51 years (ranging from 30 years to 63 years). Four patients had presented with a clinically palpable breast lump, while the fifth had presented with asymptomatic pleomorphic microcalcifications. Three patients had a suspicious mass detected on mammogram and pleomorphic microcalcifications were detected in the other 2. The diagnosis of DCIS had been made preoperatively on core or vacuum assisted biopsy in all. Possible microinvasive foci were reported in the biopsy specimen of 2 patients. The DCIS was classified as high nuclear grade in 3 patients, and necrosis was present in the biopsy specimen in 2 patients. Of the 127 patients in whom the SLN was negative for metastases, 43 patients (33.9%) were upgraded to invasive carcinoma on final histological analysis. Half of these (23 of 43) patients had undergone WLE; and 8 patients required further surgery for inadequate surgical margins. A second surgery was also required in 2 other patients who had undergone mastectomy, both of whom underwent completion ALND after the SLN was found to be positive on final histology (the SLN had not been submitted for intraoperative analysis).

An upgrade from a preoperative diagnosis of DCIS to invasive carcinoma on final histology occurred in 99 of 294 patients (33.7%). The invasive component was classified as microinvasion in 28 (28.3%) patients, as T1 disease in 57 (57.6%) patients, as T2 disease in 11 (11.1%) patients, and as T3 disease in 3 (3.0%) patients. Surgery had involved a mastectomy in 52 patients and a WLE in the other 47 patients. The regional axillary nodes had been evaluated during the initial surgery in 52 of these 99 patients, by SLN biopsy in 48 patients and by axillary sampling in the other 4. Histological upgrade occurred more frequently among women who presented with a palpable breast lump (*P* < 0.01, OR 2.60, 95% CI 1.63 to 4.16) and who had a mass detected on mammogram (*P* < 0.01, OR 2.59, 95% CI 1.53 to 4.40) or on breast ultrasound (*P* < 0.01, OR 4.22, 95% CI 1.36 to 13.07) (Table 1). Upgrade was also more likely if the preoperative diagnosis of DCIS had been made on biopsy (*P* < 0.01, OR 2.76, 95% CI 1.36 to 5.58), and particularly so if a core biopsy rather than a vacuum assisted biopsy was performed (*P* < 0.01, OR 3.77, 95% CI 1.95–7.30). Upgrade was also more likely when high nuclear grade or possible microinvasion was reported in the biopsy specimen (*P* = 0.03, OR 1.72, 0.34 to 0.98 and *P* < 0.01, OR 5.98, 95% CI 2.70 to 13.25 resp.) but was not correlated with the presence of necrosis, hormone receptor status, or tumour size (*P* > 0.05). On multivariate analysis, only a mammographically detected mass, a diagnosis made on core or vacuum assisted biopsy, and possible microinvasion in the biopsy specimen were independently correlated with the likelihood of histological upgrade to invasive carcinoma (*P* < 0.05) (Table 2).

Four patients developed axillary nodal recurrence, after a mean interval of 24.9 ± 1.77 months. Two of these patients were upgraded to invasive carcinoma following surgery and had had nodal evaluation, 1 with SLN biopsy (microinvasion found on final histology) and the other with ALND, after a 4 mm invasive ductal carcinoma was found on final histology. The nodes were negative for metastases in both and neither developed distant recurrence. The axillary nodes were not evaluated in the other 2 patients who were both managed as having pure DCIS. Distant recurrence occurred in 2 other patients who had been upgraded to invasive carcinoma. One patient had node-negative microinvasive disease (T1mic) and the other was found to have a 45 mm invasive ductal carcinoma, with 2 out of 19 nodes positive for metastasis. Both had received the recommended adjuvant treatments. There were 5 deaths during the follow-up period; 2 patients died from metastatic disease and another 3 died from nonbreast cancer related causes.

## 4. Discussion

Detecting nodal metastases in DCIS tumours can help identify the small subgroup which may benefit from systemic therapies because of a propensity for disease recurrence and metastasis [4, 5]. But although SLN biopsy can be carried out easily and with minimal morbidity, its use in all DCIS tumours is difficult to justify given that nodal metastases are so uncommon that it seldom contributes to treatment decision-making [3, 9]. Furthermore, some are now exploring whether SLN biopsy can be omitted entirely in tumours where the probability of nodal spread is minimal. Existing data cannot support this and since nodal status is a major consideration for adjuvant treatment recommendations, SLN biopsy is likely to remain the standard of care for all invasive tumours, regardless of tumour size or other tumour characteristics.

Nodal disease associated with DCIS tumours has been postulated to indicate occult microinvasion present within the primary tumour. This could possibly explain the occurrence of nodal metastases in reportedly pure DCIS tumours and may account for the more aggressive behaviour seen in a small subgroup. While exact incidence is not known, early studies have reported residual invasive disease in 10 to 20% of mastectomy specimens in women with an initial diagnosis of DCIS [10]. Occult microinvasion may have led to nodal recurrence in the 2 patients in our study with DCIS. Although the axillary nodes had not been evaluated initially, the interval between the nodal recurrence and the surgery favours recurrent disease over inadequately treated residual nodal disease. While it seems reasonable to postulate that women with tumours with occult microinvasion have an increased risk of disease recurrence and metastasis since they would not have received any systemic treatment, there are studies suggesting otherwise [11, 12]. One possible reason is that more than two-thirds of such SLN metastases are detected only on immunohistochemistry (IHC) and the prognostic significance of such occult metastases is unclear [13, 14]. In contrast to published reports, none of the patients with microinvasion in our study were found to have a positive SLN, and this is likely because H&E-negative sections were not routinely subjected to molecular techniques. Nevertheless, microinvasion was associated with disease recurrence in our study and supports the practice of evaluating the regional axillary nodes in the presence of microinvasion. The finding of nodal involvement will prompt a consideration for systemic therapy particularly when other high-risk factors are present.

In our study, the SLN was positive for tumour metastases in 3.8% of patients, and this was exclusively associated with tumours that were upgraded to invasive carcinoma following histological analysis of the surgical specimen. These support existing evidence that clinically relevant nodal disease and therefore the need for SLN biopsy are largely restricted to DCIS tumours with microinvasive or invasive foci [15]. On the other hand, no pure DCIS tumour in our study had a positive SLN, although this may not reflect the true prevalence of nodal metastases in DCIS tumours since less than half of our patients underwent SLN biopsy. Nodes were assumed to be negative in the group where the axillary nodes were not assessed, and it was reasonable to assume so since these tumours tended to have more favourable characteristics; only 27% were of high grade and median size was 9.5 mm, smaller than those where SLN biopsy was performed. But axillary recurrence did develop in 2 cases and does raise the possibility of undiagnosed axillary involvement, particularly in the one instance where nodal disease had recurred in the absence of ipsilateral breast recurrence.

The rate of histological upgrade observed in our study was relatively similar to other published studies where the preoperative diagnosis of DCIS was made on core or vacuum assisted biopsy [14, 16–21]. Core biopsy is the preferred means of preoperative diagnosis in many centres because it is minimally invasive and does not affect analysis of pathological tumour size, a major determinant of adjuvant treatment recommendations. However, invasive foci may not have been sampled or detected in the core biopsy sample because, unlike with open excisional biopsy where the bulk of the lesion is removed, tissue samples obtained from core biopsies are limited and discontinuous in nature. This can be overcome by using larger gauge needles and by sampling more cores [18, 21, 22], and our observation that upgrades were less common following vacuum assisted biopsies where larger 11-gauge needles and a greater number of cores were taken seems to support this. However, using larger needles and taking more cores potentially increase the risk of procedural complications, even if only marginally, and still will not completely eliminate the possibility of histological upgrade.

Since a proportion of tumours will be upgraded to invasive carcinoma as the preoperative diagnosis of DCIS is often made on core biopsy, these women will require repeat surgery if SLN biopsy was not performed during the initial surgery. We found histological upgrade to be more likely in women who presented with a palpable lump that corresponded to a mass on breast imaging (mammogram or breast ultrasound). Several others have also found a clinical or mammographic mass to be associated with histological upgrade [18, 19, 22–24]. That the size of the lesion should correlate with histological upgrade is reasonable since sampling error is inherent in the core biopsy technique and small invasive foci are more likely to be missed in larger lesions. A finding of high-grade DCIS in the biopsy specimen was another factor found to correlate with histological upgrade in our study. This has also been previously reported and is consistent with the frequent progression of high-grade DCIS to invasive carcinoma [25–27]. However, since high-grade DCIS is often associated with microinvasion, this likely explained why it did not remain significant in the multivariate analysis when microinvasion was included. Nevertheless, high nuclear grade would have identified additional 9 patients (9.1%) with histological upgrade in our study. Comedonecrosis and a young age at diagnosis, reported by others to be also associated with histological upgrade, were not found significant in our study [25, 28].

Identifying tumours with a high likelihood of histological upgrade will particularly benefit those women undergoing WLE. Twenty-eight percent of those who had undergone WLE in our study would have needed repeat surgery for SLN biopsy if it had not been done together with the initial surgery. Of the 23 patients who underwent WLE and SLN biopsy, 15 avoided a second surgery after invasive foci were found in the surgical specimen. Selective recommendation of SLN biopsy together with WLE will allow more surgeries to be completed in a single stage and will reduce reoperation rates. Predicting histological upgrade has less of an impact in those undergoing mastectomy since most surgeons will perform SLN biopsy at the same time. This is often because a mastectomy is often offered to those with extensive DCIS, in whom the likelihood of histological upgrade is high [25, 29]. This is not necessarily the case in our local context, where many women tend to opt for a mastectomy despite being suitable for WLE (unpublished manuscript). Many women, especially those in the older age group, perceive mastectomy as a more complete cure, with a lower risk of recurrence, and are less concerned about postoperative cosmesis. Although many of those undergoing mastectomy have in fact small tumours, we continue to routinely perform SLN biopsy together with a mastectomy since there is little added morbidity and since ALND can be avoided if nodal evaluation is required later.

## 5. Conclusion

Sentinel lymph node involvement is infrequent in women with a preoperative diagnosis of DCIS and is almost always associated with histological upgrade to invasive carcinoma. In those opting for wide local excision, findings of a mass on mammogram and possible microinvasion in the core biopsy specimen should prompt a discussion regarding the inclusion of SLN biopsy together with the initial surgery.

## Figures and Tables

**Table 1:** Correlation analyses of histological upgrade to invasive carcinoma with clinicopathological parameters (*n* = 294).

	Histological upgrade to invasive carcinoma (*n* = 99)	No histological upgrade (*n* = 195)	*P* value
Median age (years)	51 (30–91)	51 (31–85)	0.66
Ethnicity			0.09
Chinese	79	167	
Malay	13	11	
Indians	4	14	
Others	3	3	
Prior OCP/HRT use			0.10
Yes	23	30	
No	76	164	
Family history of breast cancer			0.44
Yes	16	25	
No	83	170	
Clinically palpable breast lump			<0.01
Yes	62	48	
No	73	147	
Mammographic features			<0.01
Mass	48	58	
Microcalcifications	34	114	
Architectural distortion	4	5	
Ultrasound features			<0.01
Solid mass	64	72	
No mass	4	19	
Preoperative diagnosis			<0.01
Core biopsy^†^	88	145	
Open biopsy	11	50	
Nuclear grade on biopsy			0.03
Low	10	35	
Intermediate	23	56	
High	55	88	
Presence of necrosis on biopsy			0.20
Yes	35	86	
No	53	93	
Possible microinvasion on biopsy			<0.01
Yes	23	10	
No	65	169	
Oestrogen receptor status			0.12
Positive	56	82	
Negative	31	28	
Progesterone receptor status			0.59
Positive	55	41	
Negative	54	47	
Median tumour size (mm)	12.0 (1.0 to 95.0)	13.0 (1.0 to 120.0)	0.22

OCP: oral contraceptives; HRT: hormone replacement therapy.

^†^Includes vacuum assisted biopsy.

**Table 2:** Multivariate analysis Cox regression model of the likelihood of histological upgrade to invasive carcinoma for clinicopathological parameters (*n* = 112).

	Odds ratio	Standard error	*P* value	95% confidence interval
Lump at presentation	2.81	1.55	0.06	0.95–8.30
Mass on mammogram	6.74	4.71	<0.01	1.71–26.49
Solid mass on ultrasound	2.56	2.37	0.31	0.42–15.70
Diagnosis made on core biopsy	5.82	4.80	0.03	1.16–29.30
Possible microinvasion on biopsy	17.36	15.73	<0.01	2.94–102.52
High nuclear grade on biopsy	1.13	0.56	0.81	0.42–2.99

## References

[1] Kelly T. A., Kim J. A., Patrick R., Grundfest S., Crowe J. P. (2003). Axillary lymph node metastases in patients with a final diagnosis of ductal carcinoma in situ. *The American Journal of Surgery*.

[2] Intra M., Rotmensz N., Veronesi P. (2008). Sentinel node biopsy is not a standard procedure in ductal carcinoma in situ of the breast: the experience of the European institute of oncology on 854 patients in 10 years. *Annals of Surgery*.

[3] Virnig B. A., Tuttle T. M., Shamliyan T., Kane R. L. (2010). Ductal carcinoma in Situ of the breast: a systematic review of incidence, treatment, and outcomes. *Journal of the National Cancer Institute*.

[4] Katz A., Gage I., Evans S. (2006). Sentinel lymph node positivity of patients with ductal carcinoma in situ or microinvasive breast cancer. *The American Journal of Surgery*.

[5] Sakr R., Antoine M., Barranger E. (2008). Value of sentinel lymph node biopsy in breast ductal carcinoma in situ upstaged to invasive carcinoma. *Breast Journal*.

[6] Cox C. E., Nguyen K., Gray R. J. (2001). Importance of lymphatic mapping in ductal carcinoma in situ (DCIS): why map DCIS?. *American Surgeon*.

[7] Pendas S., Dauway E., Giuliano R., Ku N., Cox C. E., Reintgen D. S. (2000). Sentinel node biopsy in ductal carcinoma in situ patients. *Annals of Surgical Oncology*.

[8] Jeevan R., Cromwell D. A., Trivella M. (2012). Reoperation rates after breast conserving surgery for breast cancer among women in England: Retrospective study of hospital episode statistics. *The British Medical Journal*.

[9] Mansel R. E., Fallowfield L., Kissin M. (2006). Randomized multicenter trial of sentinel node biopsy versus standard axillary treatment in operable breast cancer: the ALMANAC trial. *Journal of the National Cancer Institute*.

[10] Silverstein M. J., Waisman J. R., Gamagami P. (1990). Intraductal carcinoma of the breast (208 cases). Clinical factors influencing treatment choice. *Cancer*.

[11] Fisher B., Dignam J., Wolmark N. (1998). Lumpectomy and radiation therapy for the treatment of intraductal breast cancer: findings from National Surgical Adjuvant Breast and Bowel Project B- 17. *Journal of Clinical Oncology*.

[12] Murphy C. D., Jones J. L., Javid S. H. (2008). Do sentinel node micrometastases predict recurrence risk in ductal carcinoma in situ and ductal carcinoma in situ with microinvasion?. *The American Journal of Surgery*.

[13] Klauber-DeMore N., Tan L. K., Liberman L. (2000). Sentinel lymph node biopsy: is it indicated in patients with high-risk ductal carcinoma-in-situ and ductal carcinoma-in-situ with microinvasion?. *Annals of Surgical Oncology*.

[14] Wilkie C., White L., Dupont E., Cantor A., Cox C. E. (2005). An update of sentinel lymph node mapping in patients with ductal carcinoma in situ. *The American Journal of Surgery*.

[15] Huo L., Sneige N., Hunt K. K., Albarracin C. T., Lopez A., Resetkova E. (2006). Predictors of invasion in patients with core-needle biopsy-diagnosed ductal carcinoma in situ and recommendations for a selective approach to sentinel lymph node biopsy in ductal carcinoma in situ. *Cancer*.

[16] Lee C. H., Carter D., Philpotts L. E. (2000). Ductal carcinoma in situ diagnosed with stereotactic core needle biopsy: can invasion be predicted?. *Radiology*.

[17] Pandelidis S., Heilman D., Jones D., Stough K., Trapeni J., Suliman Y. (2003). Accuracy of 11-gauge vacuum-assisted core biopsy of mammographic breast lesions. *Annals of Surgical Oncology*.

[18] Chan M. Y., Lim S. (2010). Predictors of invasive breast cancer in ductal carcinoma in situ initially diagnosed by core biopsy. *Asian Journal of Surgery*.

[19] Goyal A., Douglas-Jones A., Monypenny I., Sweetland H., Stevens G., Mansel R. E. (2006). Is there a role of sentinel lymph node biopsy in ductal carcinoma in situ? Analysis of 587 cases. *Breast Cancer Research and Treatment*.

[20] Dillon M. F., McDermott E. W., Quinn C. M., O'Doherty A., O'Higgins N., Hill A. D. K. (2006). Predictors of invasive disease in breast cancer when core biopsy demonstrates DCIS only. *Journal of Surgical Oncology*.

[21] Park A. Y., Gweon H. M., Son E. J., Yoo M., Kim J.-A., Youk J. H. (2014). Ductal carcinoma in situ diagnosed at US-guided 14-gauge core-needle biopsy for breast mass: preoperative predictors of invasive breast cancer. *European Journal of Radiology*.

[22] Brennan M. E., Turner R. M., Ciatto S. (2011). Ductal carcinoma in situ at core-needle biopsy: meta-analysis of underestimation and predictors of invasive breast cancer. *Radiology*.

[23] Meijnen P., Oldenburg H. S. A., Loo C. E., Nieweg O. E., Peterse J. L., Rutgers E. J. T. (2007). Risk of invasion and axillary lymph node metastasis in ductal carcinoma in situ diagnosed by core-needle biopsy. *British Journal of Surgery*.

[24] Ciatto S., Houssami N., Ambrogetti D. (2007). Accuracy and underestimation of malignancy of breast core needle biopsy: the Florence experience of over 4000 consecutive biopsies. *Breast Cancer Research and Treatment*.

[25] Yen T. W. F., Hunt K. K., Ross M. I. (2005). Predictors of invasive breast cancer in patients with an initial diagnosis of ductal carcinoma in situ: A guide to selective use of sentinel lymph node biopsy in management of ductal carcinoma in situ. *Journal of the American College of Surgeons*.

[26] Page D. L., Dupont W. D., Rogers L. W., Landenberger M. (1982). Intraductal carcinoma of the breast: follow-up after biopsy only. *Cancer*.

[27] Collins L. C., Tamimi R. M., Baer H. J., Connolly J. L., Colditz G. A., Schnitt S. J. (2005). Outcome of patients with ductal carcinoma in situ untreated after diagnostic biopsy: results from the nurses' health study. *Cancer*.

[28] Hardman P. D. J., Worth A., Lee U. (1989). The risk of occult invasive breast cancer after excisional biopsy showing in-situ ductal carcinoma of comedo pattern. *Canadian Journal of Surgery*.

[29] Tan J. C. C., McCready D. R., Easson A. M., Leong W. L. (2007). Role of sentinel lymph node biopsy in ductal carcinoma-in-situ treated by mastectomy. *Annals of Surgical Oncology*.

